# A Novel Framework for Fog-Assisted Smart Healthcare System with Workload Optimization

**DOI:** 10.1155/2022/4174805

**Published:** 2022-09-29

**Authors:** Ahmed A. H. Abdellatif, Aman Singh, Abdulaziz Aldribi, Arturo Ortega-Mansilla, Muhammad Ibrahim

**Affiliations:** ^1^Department of Pharmaceutics, College of Pharmacy, Qassim University Buraydah, Buraydah, Saudi Arabia; ^2^Prince Faisal Bin Mishaal Artificial Intelligence Chair, Qassim University, Buraydah, Saudi Arabia; ^3^Higher Polytechnic School, Universidad Europea del Atlántico, C/Isabel Torres 21, Santander 39011, Spain; ^4^Department of Computer Science, College of Computer, Qassim University, Buraydah, Saudi Arabia; ^5^Department of Project Management, Universidad Internacional Iberoamericana, Arecibo 00613, Puerto Rico, USA; ^6^Department of Project Management, Universidad Internacional Iberoamericana, Campeche 24560, Mexico; ^7^Department of Information Technology, University of Haripur, Haripur 22620, Pakistan

## Abstract

Fog-assisted and IoT-enabled smart healthcare system with rapid response rates is the major area of concern now a days. Dynamic and heterogeneous fog networks are difficult to manage and a considerable amount of overhead could be realized while managing ever increasing load on foglets. Fog computing plays a vital role in managing ever increasing processing demands from diverse IoT-based applications. Smart healthcare systems work with the assistance of sensor-based devices and automatic data collection and processing can speed up overall system functionality. In the proposed work, a novel framework for smart health care is presented where a series of activities are performed with prime objective of reducing latency and execution time. Principal component analysis is used for feature reduction and support vector machines with radial basis function kernel is used for classification purpose. Workload optimization on the fog nodes is implemented using genetic algorithm. Data collection process also involves preprocessing as a leading step for generating cleaner data. Amalgamation of intelligent and optimization techniques in the presented framework certainly improves the efficiency of the overall system. Experimental results reveal that proposed work outperforms the existing fog-assisted smart healthcare systems in terms of latency, execution time, overall system accuracy, and system stability.

## 1. Introduction

With the emergence of IoT devices and related technology, smart healthcare systems have evolved tremendously. The existence of these systems and their associated benefits have also paced up the processing speed, and the accuracy levels of the results could be seen on a higher side. Fog computing has also emerged as a supporting technology in this dynamic era to fulfill the current requirements. It serves as an intermediate between the cloud and the IoT layer [1]. Intelligent healthcare systems are latency-sensitive, and sending all the requests to the cloud layer can cause significant delays. The fog layer solves the above-stated problem, and non-critical tasks could be served immediately so that only critical tasks could be sent to the cloud layer. Cloud layer can provide resources in large amounts, which could be required to process critical tasks. Processing of non-critical tasks at the fog layer can reduce a considerable amount of overhead on the cloud layer [2].

Fog computing paradigm works by processing the tasks generated at the IoT layer. As, this layer works as the middle layer, it can produce results quickly which could be used by another set of operations. Fog servers possess less resources, due to which it becomes difficult to manage large set of tasks. For resolving this issue, proper task management should be applied with suitable optimization procedures. Fog computing offers its assistance in a diversified set of applications like: Smart City, Smart Grid, Smart Industry, Smart healthcare etc., With the increasing attention towards healthcare sector, fog computing is playing a pivotal role and also is proving its effectiveness in this area. In the last couple of years, enormous amount of research has been laid on smart healthcare with promising results.

**Figure 1:**
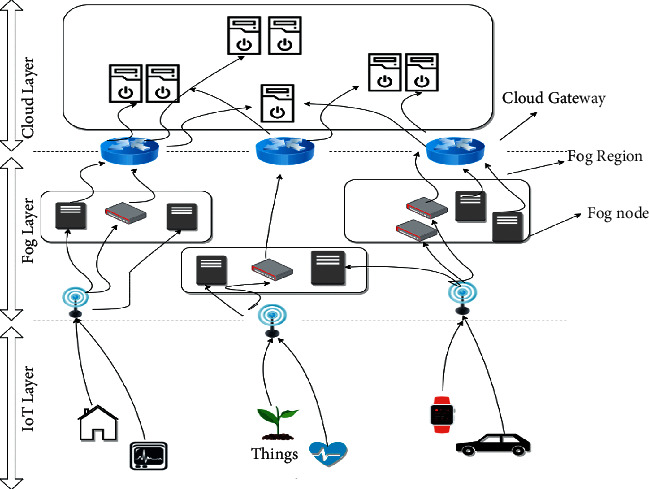
Three-tier mechanism of fog computing [1].

Fog computing enables location awareness, support for user accessibility, real-time interactions, low latency, high scalability, and interoperability, which cloud computing systems could not support. Because most of the information is being processed locally in the fog nodes, only summarized data will be transported to the cloud, resulting in significant bandwidth savings. It will decrease latency, delays, and packet loss [3]. Fog computing requires effective resource management. The three-tier mechanism of fog computing is presented in Figure 1.

Managing workload in the fog computing paradigm is a major challenge, as numerous requests are generated from the IoT layer at the same point of time. Timely management of such requests becomes crucial because of the limited number of resources at the fog layer. In addition to it, some concern must be given to critical smart healthcare systems as well, where delay could not be tolerated because of lack of load management. This issue could further put a negative impact on the related performance parameters such as: response time, energy consumption, cost. Proper load balancing works to accomplish multiple objectives such as: optimal throughput and minimized response time which further improves latency and energy consumption [4].

### 1.1. Limitations and Research Goals

This research work is the extension to the work that has been done over the years in the field of smart healthcare systems. Various limitations from the existing systems are extracted, and they all can be summarized as following [1, 2, 4]:

- lack of data preprocessing—large amount of sensor data is generated which is required to be preprocessed before actually going for the final prediction and analysis.
- Heavy workload on fog servers—as sensors can generate various tasks and huge amount of data is required to be processed, fog servers could be easily overloaded, and it can hamper their performance and latency could be achieved.
- Heavy energy consumption—due to overloaded fog servers, there could be a chance of heavy energy consumption which can reduce the overall system efficiency.
- High Latency—improper data processing and resource management can cause considerable amount of delays, therefore high latency could be observed.
- Dynamic requests—smart environments are highly dynamic in nature and sometimes it becomes tedious task to manage the ever-changing requests, as fog servers are mostly configured for the static set of requests from the IoT layer.
- Unnecessary features for task classification—there are various features or data attributes which are unnecessary and they play a minimal role in the final classification of the task category.

The above section is signifying the need of extending the existing research work to formulate new procedures which can minimize the effects of identified limitations. A summarized view of various objectives with respect to desired architecture for smart healthcare system is presented below. The first objective is to perform data preprocessing to achieve cleaner and accurate data. The second objective is to minimize workload on the overloaded fog servers which can lead to fulfillment of the third objective of reducing latency and lastly it can handle dynamic requests from the IoT layer. The abovementioned objectives are achieved using the following phases of designed architecture, such as:

- implementation of data preprocessing techniques for noise removal and data filtering.
- Application of dimensionality reduction technique: principal component analysis (PCA) for reducing unwanted features.
- Classification of tasks as critical and non-critical using SVM (support vector machine) for performing the processing at cloud and fog layers.
- Managing load allocation on the fog servers using nature-inspired optimization algorithm, i.e., genetic algorithm

These phases work in a repetitive manner to achieve the desired goals and to provide solutions to the abovementioned problems. The rest of the paper is organized like, Section 2 is focused on the existing work with respect to smart healthcare systems.  Section 3 is focused on the proposed work and the methodology to achieve the desired research goals. Experimental settings and results are discussed in Section 4, and conclusion part sums up the entire work in Section 5.

## 2. Related Work

Over the years, noticeable research is performed to streamline various operations of smart healthcare systems. Multiple approaches are designed with fog and cloud layers to reduce the service delay and enhance the processing capabilities. For instance, framework using ensemble deep learning is developed for automatic heart disease analysis. Performance of the proposed system is evaluated using various parameters, and simulation is performed with the help of FogBus. The proposed work was tested on the real-time heart patient dataset, and good accuracy levels were observed. It considers various parameters for validation purpose and therefore successfully overpowers the existing systems [5]. A personalized healthcare model was also designed with the help of dynamic programming approach for providing quick service as per patient's health status. The overall model is known as smart treatment for personalized healthcare which optimizes the healthcare services through intelligent agents [6]. Some hybrid approaches were also designed as a solution to the problems incurred in the cloud-only systems. Features of naïve Bayes and firefly algorithms were combined to perform the health prediction of elderly patients [7].

Gait-based system for older adults was presented where acceleration-based gait recognition method was implemented for increasing the recognition performance of IoT-based devices. It uses PCA (principal component analysis) technique for dimensionality reduction which further helps in the identity recognition. The mentioned IoT devices are the wearable devices for the patients which further sends the patient's data to the cloud layer for processing [8]. Real-time health-monitoring system was also presented based on the four-level remote triage and package localization. A set of 12 hospitals were selected for analysis, and later their ranking was performed. The major parameter for ranking was based on the services provided with respect to healthcare. Various types of sensors were deployed to receive patient's data, and the dataset was formulated with a variety of symptoms [9]. Reversed naive Bayesian logic was also implemented to enhance the classification accuracy of biomedical data. The major aim was to ignore those data instances which can reduce the classification accuracy. So, after reducing the irrelevant data instances, identified noise instances were used for training the model. This approach can reduce the overall training effort to a large extent, and biomedical data classification could be achieved. Various datasets from UCI were taken for the validation purpose and promising results were seen [10].

Cloud-based health care system was designed which used the concept of digital twin. This approach helps in performing medical simulations for achieving faster and accurate results. It works in three parts, such as physical and virtual objects followed by healthcare data. All medical devices are the part of physical object, whereas virtual object focuses on modelling of medical devices. Healthcare data is the outcome from the sensors and wearable devices. This technique can decrease efforts required for real-time supervision, and the overall system accuracy could be enhanced [11]. Medical data monitoring system was also presented for sensor fault detection. The main objective of this system was to identify those sensor nodes which can generate false results. So, for achieving this, a Bayesian network model was designed based on conditional probability distribution. After implementing the proposed idea, fault detection accuracy increased to a greater extent [12].

Another system which deployed hierarchical computing architecture and convolutional neural network (CNN) was presented which works with an objective of real-time monitoring of patients and performs classification on the ECG data. The main purpose of this work was to enhance the performance accuracy by exploiting the deep learning methods on a real-time dataset. Parameters such as: response time and accuracy were chosen for the validation of the proposed work, and promising results were observed [13]. A novel cloud-based healthcare system was presented with prime purpose to monitor health and perform diagnosis. Cloud layer was presented to perform logic processing using deep-learning-based architecture. CNN-based model was used to achieve desired results. Data preprocessing mechanism was also implemented to assist the working methodology of proposed system [14].

Fog-assisted health monitoring system was presented which used the concept of temporal mining to generate the health index for a patient. It used Bayesian belief network classifier to train the system. Dataset of various users in smart homes was constructed and variety of parameters were involved to test the accuracy of the proposed system. It was also capable of generating alarms in critical situations [15]. A fog-cloud-computing-based architecture was presented, where user health was monitored in smart office environment. It used the severity index to keep track of the health and generated alerts if situation degrades. It focused on accumulating data from various sources like: health, environment, meal, physical postures. Comparison was also performed with various baseline algorithms such as: K-NN, ANN, and SVM, and the proposed work based on probabilistic measures outperformed them [16].

Fog-assisted healthcare system was proposed, which combined medical signals and sensor data to formulate a remote pain monitoring system. This work aimed to reduce latency and network consumption and validate the effectiveness of proposed work after comparing with cloud-based systems. The two types of signals, sEMG and ECG, were processed to compute the pain levels of the patients. Authors have justified the usage of fog-computing-based architecture in context of computations and storage on the edge layer which is closer to the biomedical sensors, hence producing timely results [17]. For managing heterogeneous fog computing environment and inconsistent communication between fog and the edge devices, an efficient approach was presented where application placement could be done on the network nodes. It tried to improve latency and final network consumption [18]. Another approach was designed for mapping sensor nodes to its parent fog node effectively, so that overall system performance could be improved. Authors designed this approach to handle varied computing capabilities of fog nodes and look to reduce delay and improve network utilization [19].

Another healthcare system based on complex event processing was presented where data sources were combined at certain intervals to generate timely responses. The major objective was to obtain a higher response rate as compared to the traditional healthcare systems [20]. Tri-fog health [21] was also presented which used a series of techniques to overpower the existing healthcare systems. It eliminated the faulty data in the wearable layer itself. It also performed redundant data elimination using FaMOORA algorithm. Furthermore, it used SpikeQ-Net for training the system. It was implemented as a hybrid machine learning algorithm. It also performed fog offloading using multi-objective spotted hyena optimization algorithm. As it performed a series of activities in wearable and fog layer, this architecture had provided promising results.

After going through related work, various strengths and limitations were identified and their summary is presented in Table 1.

## 3. Proposed System Framework

In this section, the proposed framework is defined which works in four phases to perform the required set of actions.

### 3.1. Problem Statement

Smart healthcare systems generate numerous amounts of tasks and heavy load which is required to be processed frequently, so that, instant decisions could be taken. Over the years, many computational architectures were proposed with an objective of reducing latency and execution time. But these systems were lacking refinements and optimizations at certain levels. This proposed work was directed to solve stated problem where every layer in the three-tier architecture was intended to do some tasks; hence, overall system was regulated for achieving desired goals. Moreover, heterogeneous fog nodes and varied computational capabilities were difficult to manage, so, constant monitoring and regular optimizations were required which can improvise overall system's efficiency.

### 3.2. System Overview

The proposed system framework is designed to work in three layers, i.e., the IoT layer, the fog layer, and the cloud layer. The IoT layer consists of various types of sensors for collecting the medical data which is preprocessed for removing noise and various outliers. Final preprocessed data is passed on to the set of fog nodes (F1, F2, F3…) where dimensionality reduction procedure works and only necessary features are extracted. This step reduces the effort and overall performance of the fog layer is enhanced. The fog layer also implements the task classification technique for categorizing critical and non-critical tasks. Furthermore, optimization method is also incorporated for overloaded fog nodes. For instance, if any fog node is receiving bulk of requests, that could be offloaded and forwarded to the idle foglets.

**Figure 2:**
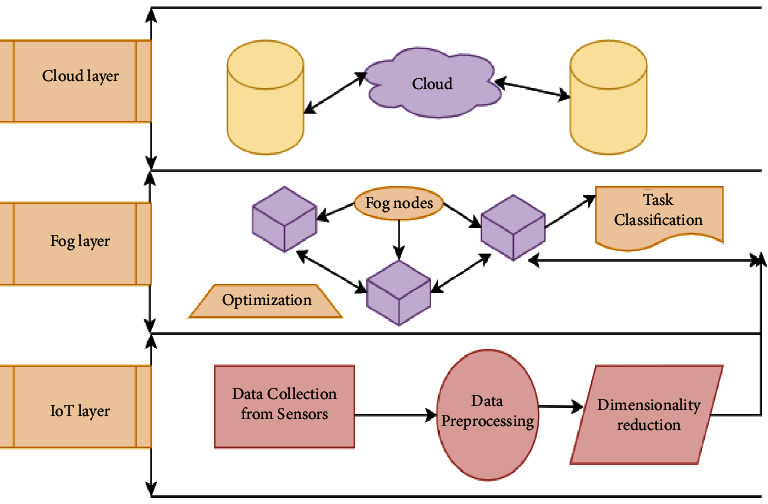
Overview of the proposed system.

Once fog layer is done with its all functionalities, the critical tasks are sent to the cloud layer for the final processing and the responses are generated for necessary action. Overall system architecture could be implemented for variety of scenarios, but it will be more suitable for the healthcare systems where even the minor delays are not acceptable and responses should be processed rapidly.  Figure 2 is demonstrating the same scenario discussed above. Each layer plays its individual role and moreover series of functions are performed as per the requirement of the proposed architecture.  Table 2 highlights the series of steps performed in each phase along with the required technique implemented for the same.

### 3.3. System Description

A brief overview of the proposed system is presented in Figure 2. Every layer in the proposed architecture consists of a series of activities which can play their part in increasing the overall system performance.  Table 2 demonstrates the specific set of techniques used in each layer for implementing the proposed methodology.

From Table 2, we can see the list of activities performed in each layer, along with that, the techniques and resource requirement are also mentioned. Every technique or mentioned algorithm is customized as per the requirement of a smart healthcare system. Following subsections are covering the complete details of these activities.

### 3.4. Data Collection

This is the initial step where patient data from various biomedical sensors will be collected and dataset will be formulated. Common parameters, such as blood pressure, temperature, heart rate, Oxygen levels, will be considered, and dataset will be updated after regular intervals. The collected data will be maintained in a file which will be populated with the parameter values on regular basis. Repository of dataset will be maintained online.

### 3.5. Data Preprocessing

The automated data collection process can gather noisy data which is required to be cleaned by using some procedures, such as filling up of missing values, identification of outliers, and removing inconsistent values. Routines for performing these all these steps are written and cleaned data is sent for further processing. Sensor data could be easily misunderstood by the receiving algorithm and misclassification could be done. Moreover, various outliers in the data can produce false warnings, and overall system performance will be dropped. Some missing values could also be observed, if due to some reasons, any sensor has stopped working or it is damaged. In these cases, it would be better to have the average values, rather than going for blank set of values. Inconsistent data patterns could also be experienced with faulty sensors, so there should be a mechanism to identify those patterns and look for some countermeasures.

### 3.6. Dimensionality Reduction

This is one of the crucial steps of the methodology wherein only the most important features are extracted and unwanted features are dropped. This step can reduce a considerable amount of effort incurred, and overall system efficiency could be enhanced. PCA (principal component analysis) technique is used to perform this task.

#### 3.6.1. Principal Component Analysis Model

It is a multivariate technique whose major role is to analyse the provided data table and to identify the principal components from it. PCA focuses on reducing the number of features without actually reducing the accuracy levels. The major objective is to remove the extraneous variables which can hamper the overall performance. In the proposed system also, this technique is applied to reduce the extraneous medical features, so that rapid classification could be done. It works in a series of steps, such as the first step is to provide standardization for the initial variables. This is done to reduce the variance in the data values. After this step, a covariance matrix is computed to analyse the correlation among various variables. The next step involves the computation of eigen values and vectors to give away the principal components. This step is followed by another step where feature vector is generated. All principal components are not significant, so it becomes necessary to discard some of them. Only significant PCs are taken into consideration to generate the final feature vector. Following equation denotes the final dataset which consists of a reduced number of features decided as per selected eigen vectors [22].

(1) $$\begin{array}{c} FD={FV}^{P}\;*\;{SOD}^{P}\text{,} \end{array}$$

FD: final dataset, FV: feature vector, SOD: standardized original dataset.

Major tasks of PCA involves the extraction of key information from the dataset and to simplify the complex data description. It can also analyse the patterns of observations and variables associated with them. The extracted information is in the form of principal components which are also known as orthogonal variables. The task of achieving necessary information/or lower dimensions is achieved using transformation of given data, i.e.,

(2) $$\begin{array}{c} Y=\left\{y_{1},y_{2},\ldots \ldots ,y_{N}\right\}\text{.} \end{array}$$

from S^A^[Higher dimension] to S^B^[Lower dimension]. In (2), N denotes the maximum number of instances, and *y*_i_ denotes i^th^ instance. Further, principal components are derived, and every component has different levels of data variance. For example, the very first component will exhibit maximum data variance, whereas the second one represents next maximum variance and the process of principal components computation continues. There are various methods of calculating principal components such as: covariance-matrix-based and singular-value decomposition. The proposed framework implements covariance-matrix-based methodology to calculate principal components and furthermore dimensionality reduction could be achieved. Covariance matrix (equation (3)) represents various essential things, such as the diagonal elements represent variance among two variables and further if there is a positive element available in the matrix, it reflects the positive correlation and a negative value will highlight the negative correlation. A zero value represents there is no correlation among the two variables. Once the covariance matrix is formulated the next task is to compute the eigen values and vectors.

Eigen vectors are always non-zero vectors and each individual vector represents the principal component. Eigen values are of scalar type and eigen vector with the highest eigen value represents the first principal component, and further principal components are decided as per eigen values in the descending order.

(3) $$\begin{array}{c} \left(\begin{array}{c} V\left(y_{1},y_{2}\right),C\left(y_{1},y_{2}\right)\ldots \ldots C\left(\left(y_{1,}y_{Z}\right)\right.\\ C\left(y_{2},y_{1}\right),V\left(y_{2},y_{2}\right)\ldots \ldots C\left(y_{2,}y_{Z}\right)\\ ---------------\\ C\left(y_{z},y_{2}\right),C\left(y_{z},y_{2}\right)\ldots \ldots V\left(y_{z,}y_{Z}\right) \end{array}\right)\text{.} \end{array}$$

The major task while calculating eigen vectors and values is to maximize the variance. It can be done through stacking *p* data vectors into *p* × *q* matrix, a, then, the projections are given by ab, which is an *p* × 1 matrix [22]. Variance could be computed as:

(4) $$\begin{array}{c} \sigma_{\overset{⟶}{b}}^{2}\frac{1}{p}*\left(\underset{i}{\sum }{\left(\overset{⟶}{a_{i}\;}.\overset{⟶}{b\;}\right)}^{2}\right)=\frac{1}{p}*\left({\left(ab\right)}^{T}\left(ab\right)\right)=\frac{1}{p}*\left(b^{T}a^{T}ab\right)=b^{T}*a^{T}\frac{a}{p}*b=b^{T}vb\text{.} \end{array}$$

It is desirable to select a unit vector $\overset{⟶}{b}$ so that there is maximum $\sigma_{\overset{⟶}{b}}^{2}$. For doing so, unit vectors must be analyzed so that maximization is constrained. After successfully implementing the above strategy, the maximum variance can be obtained, and it can further help in finding the best possible eigen vectors for computing principal components. We can conclude that there is v matrix with dimensions *k* × *k* where *k* denotes different eigen vectors. Eigen vectors are always orthogonal to each other. These computed eigen vectors are also known as principal components of data. The first principal component is one, which is having the maximum data variance, where the next set of principal components are decided as per next levels of data variance. Algorithm 1 is specifying the overall process for dimensionality reduction and extracting essential features from the entire set.

Here, ∑**a** denotes the covariance matrix from which principal components will be generated, and overall data *Z* will be projected into PCA subspace after calculating eigen values and vectors.

### 3.7. Task Classification Using Support Vector Machines (SVM, RBF-Kernel Based)

Task classification is one of the major steps involved which decides on whether the submitted request in the form of a series of data values is critical or non-critical. For doing so, supervised machine learning algorithm is implemented with the help of radial basis function kernel. SVM can be used for both classification and regression analysis. Here, it is implemented for the classification problem. SVM works with the principal of finding hyperplane that can isolate the features into classes. Kernels can play a major role in high dimensional data. Here, in the proposed work, data of numerous features is collected which is used for further system training. It becomes necessary to implement the kernel trick for getting a better system accuracy. Radial basis function kernel is also known as Gaussian-based kernel which is primarily used for non-linear data. Following equation determines the mathematical model of RBF-based kernel.

(5) $$\begin{array}{c} A\left(P,P^{'}\right)=\exp\;\left(-gamma*{\left(\left\|P-P'\right\|\right)}^{\left(2\right)}\right) \end{array}$$

Here, gamma value can vary from 0 to 1. Correct usage of gamma value plays an important role. Values such as 0.1 is the most preferred value to avoid underfitting and overfitting of the data. A dataset with *p* no. of samples is denoted as: $\left\{\overset{⟶}{Z_{j}},n_{j}\right\}$,*j* = 1,2,……,p with $\overset{⟶}{Z_{j\;}}\in {\left\{0,1\right\}}^{x}$ exhibits sample data and **n**_**j**_∈ {+1,-1} denotes class label of given sample. For a testing sample *Z*, RBF-kernel-based SVM classification formula is as follows:

(6) $$\begin{array}{c} F\left(Z\right)=signfunc\left(c+\overset{p}{\underset{j=1}{\sum }}\gamma_{j}n_{j}\;K\left(\overset{⟶}{Z_{j}},Z\right)\right)\text{.} \end{array}$$

In the given equation *γ*_**j**_ and *c* are knowledge factors of SVM and given signfunc() is the sign function whereas $K\left(\overset{⟶}{Z_{j}},Z\right)$ represents a kernel function [24]. Projection of RBF kernel is also done into infinite dimensions, such as:

(7) $$\begin{array}{c} A\left(P,P'\right)=<\varphi \left(P\right),\varphi \left(P^{\prime }\right)\text{.} \end{array}$$

Here, *φ*, is a type of function, that projects vectors *y* into another vector space. This function also calculates inner-product between two vectors. It can also project vectors into infinite dimension and proof for the same is also provided as per following:

(8) $$\begin{array}{c} \varphi_{IRBF}=Kn⟶K^{\infty }\text{.} \end{array}$$

Consider gamma value in equation (5) to be ½,

(9) $$\begin{array}{c} AIRBF\left(P,P'\right)=\exp\;\left[-\frac{1}{2}|{\left.\left|P-P^{\prime }\right|\right|}^{2}\right]\\ =\exp\;\left[-\frac{1}{2}<P-P^{\prime },P-P^{\prime }>\right]\\ =\exp\;\left[-1/2\left(\langle P,P-P^{\prime }>-<P,P-P^{\prime }\rangle \right)\right]\\ =\exp\;\left[-\frac{1}{2}\left({\left|\left|P\right|\right|}^{2}+{\left|\left|P^{\prime }\right|\right|}^{2}-2<P,P^{\prime }>\right)\right]\\ =\exp\;\left[-\frac{1}{2}\left({\left|\left|P\right|\right|}^{2}\right.-\frac{1}{2}{\left|\left|P^{\prime }\right|\right|}^{2}\right]\exp\;\left[-\frac{1}{2}-2<P,P^{\prime }>\right]\\ =Ge^{\langle P,P^{\prime \;}\rangle },Here\;G\;is\;constant\\ =G\overset{\infty }{\underset{n=0}{\sum }}\;<P,P^{\prime }>n/n!\\ =G\overset{\infty }{\underset{n=0}{\sum }}\;A_{poly\left(n\right)}\left(P,P^{\prime }\right)/n!. \end{array}$$

Equation (9) proves that RBF kernel is formed by performing infinite sum over polynomial kernels. Consider the sum of two kernels, such as:

(10) $$\begin{array}{c} \begin{array}{c} Az\left(P,P'\right)≔Ax\left(P,P'\right)+Ay\left(P,P'\right)\text{,}\\ \varphi_{z}\left(P\right)=\left(\varphi_{x}\right.\left(P\right)+\varphi_{\left.y\left(P\right)\right)}\text{.} \end{array}\text{.} \end{array}$$

Here, *φ*_**z**_(P) is a tuple, where the first element is the vector *φ*_**x**_(P), and the other element is *φ*_**y**_(P). Inner product is given by:

(11) $$\begin{array}{c} \langle \varphi_{z}\left(P\right)\varphi_{z}\left(P^{\prime }\right)\rangle ≔\langle \varphi_{x}\left(P\right)\varphi_{x}\left(P^{\prime }\right)\rangle +\langle \varphi_{y}\left(P\right),\varphi_{y}\rangle \left(P^{\prime }\right)\text{.} \end{array}$$

In general, it is given as:

(12) $$\begin{array}{c} <\varphi_{z}\left(P\right),\varphi_{z}\left(P'\right)>≔\overset{di\;mension\left(x\right)}{\underset{i}{\sum }}\varphi_{x,i\;}\left(P\right)\varphi_{x,i\;}\left(P^{\prime }\right)+\overset{di\;mension\left(x\right)}{\underset{j}{\sum }}\varphi_{x,j\;}\left(P\right)\varphi_{y,j\;}\left(P^{\prime }\right)\text{.}\\ =\overset{di\;mension\left(x\right)+di\;mension\left(y\right)}{\underset{i}{\sum }}\varphi_{z,i\;}\left(P\right)\varphi_{z,i\;}\left(P^{\prime }\right) \end{array}$$

Finally, this is the projection into a vector space with infinite dimension.

### 3.8. Workload Optimization Using Genetic Algorithm

Due to heavy network traffic, there is a certain chance that a couple of fog nodes may get overloaded. So, it becomes necessary to perform the optimization after certain interval of time. Workload optimization procedure will try to locate the idle fog nodes. Once the idle fog nodes are identified, fog offloading could be implemented and various requests could be migrated from overloaded foglet. For doing so, genetic algorithm (GA) is implemented as a part of the working methodology. Genetic algorithm works on the principal of natural evolution. It works by choosing the fittest population from the given set. Firstly, the parents with the best surviving capabilities are identified, and later, their offspring will come, which could be better as compared to their parents. This process keeps on happening, and at the end, the best population set is achieved. It works in five phases such as: (a) selection of initial population, (b) fitness function, (c) selection, (d) crossover, and (e) mutation [25].

- So, it starts by choosing the initial populations where genes which characterizes an individual are selected to form chromosomes, and by doing so, the final population is decided. In the proposed system also, initial set of fog nodes are decided, such as: F1, F2, F3, F3…
- The next step is to choose a fitness function which determines the ability of an individual to survive with the other set of individuals available in the system. So, for doing so, fitness score will be calculated for the individual. Same scenario is simulated for the available fog nodes, and their fitness scores are calculated
- After selecting the fittest individuals, routines will be designed to pass on the genes (or acquired features) into the next generations to obtain the optimized results
- next step is to perform crossover, where crossover points are decided up to which parents can keep on sharing genes and offspring is generated.
- last step is to perform mutation where certain changes are performed in the genes of offspring to bring versatility in the generated population.

All steps mentioned above use the concept of survival of the fittest. Genetic operators will be implemented on the list of individuals to generate new population. Following is the mathematical representation of various steps involved in GA optimization process:

Consider initial population(Z) of *x* chromosomes. The first task is to calculate fitness value of every chromosome. Consider there are two chromosomes CH1 and CH2 chosen from the overall population as per their respective fitness values. There is a crossover probability associated with every chromosome, and same is applied on CH1 and CH2 to produce new offspring, such as Osp. Once offspring (Osp) is generated, it is correlated with mutation probability to generate new offspring (NOsp). This newly generated offspring will be a part of the new population. The process of selection, crossover, and mutation will be executed again and again until unless the new population is completely formulated. The existing search process of GA is continued to achieve the optimal solution. The global search capabilities of GA are better as it can assess numerous individuals, and hence, a variety of optimal solutions could be generated. Crossover is the vital process which will generate the offspring, and it is denoted as:

(13) $$\begin{array}{c} X=\frac{\left(P+2\sqrt{s}\right)}{3P}\text{.} \end{array}$$

Here, s is the number of generations, P is the total number of evolutionary generation sets by population. *X* is dynamic in nature, and it can vary, as the number of evolutionary generations can also change with constant intervals of time. Complete specification of the optimization process is denoted by Algorithm 2.

Following steps are giving the complete details of fitness function and supporting equations with respect to modified GA for workload optimization:

(a) Total no. of individuals at every time step *x* and can satisfy *Z* are given as:
  (14) $$\begin{array}{c} I_{z,x}=|Yx\;\left.\cap Z\right|\text{.} \end{array}$$
(b) Following expression provides observed average fitness at time *x*:
  (15) $$\begin{array}{c} \bar{a\left(x\right)=\left.\frac{1}{n}*\overset{n}{\underset{i=1}{\sum }}a\left(q_{i}\right.,x\right).} \end{array}$$
(c) Term $\bar{a}$(Z,x) means schema's observed average fitness, where schema is denoted by *Z*, at time *x*, such as:
  (16) $$\begin{array}{c} \bar{a\left(Z,x\right)=\frac{1}{IZ,X}\;*\;\sum_{i\in \left\{j\right)|q_{j,x\in z}}a\left(q_{i},x\right).} \end{array}$$

Following inequality holds for every schema Z

(17) $$\begin{array}{c} E\left[Iz,x+1\right]>=Iz,x.\bar{a}\left(Z,x\right)/\;\bar{a}\left(x\right).\frac{\left(1-{\mathrm{pr}}_{c}-\gamma \left(Z\right.\right)}{n-\left.1\right).{\left(1-{\mathrm{pr}}_{m}\right)}^{\alpha \left(H\right)}}\text{.} \end{array}$$

Probability of choosing an individual, satisfying *Z* is:

(18) $$\begin{array}{c} \left.\frac{\sum_{i\in \left\{j\right)|q_{j,x\in z}}a\left(q_{i},x\right)}{\sum_{i=1}^{n}a\left(q_{i}\right.,x}\right)\text{.} \end{array}$$

Given that the probability is a constant and remains the same in complete iterations of the loop. To obtain desired number of selected individuals satisfying *Z* (with sample amount: n),

(19) $$\begin{array}{c} \begin{array}{c} n.\;\sum_{i\in \left\{j\right)|q_{j,x\in z}}a\frac{\left(q_{i},x\right)}{\left.\sum_{i=1}^{n}a\left(q_{i}\right.,x\right)}=n.\;Iz,x/Iz,x\;.\;\sum_{i\in \left\{j\right)|q_{j,x\in z}}a\frac{\left(q_{i},x\right)}{\left.\sum_{i=1}^{n}a\left(q_{i}\right.,x\right)}\\ =Iz,x\;.\;\bar{a}\left(Z,x\right)/\;\bar{a}\left(x\right) \end{array}\text{.} \end{array}$$

If there are two individuals crossed, then, the probability that the cross site is selected within the given length of *Z* is:

(20) $$\begin{array}{c} \alpha \left(Z\;/\;n-1\right) \end{array}$$

Survival probability Pr_s_ of Z:

(21) $$\begin{array}{c} \alpha \left(Z\;/\;n-1\right) \end{array}$$

Here, Pr_c_ is the crossover probability.

After executing selection and crossover, we can compute no. of strings satisfying *Z* after crossover using

(22) $$\begin{array}{c} \bar{a\left(Z,x\right)/\;}\bar{a}\left(x\right).\;Iz,x.\;\mathrm{Pr}\;s>=\bar{a\left(Z,x\right)/\;\bar{a}\left(x\right).Iz,x\;.\;\left(1\right.-\mathrm{Pr}\;c.\;\alpha \left(Z\;/\;n-1\right).} \end{array}$$

Probability that all specifications of *Z* are unaffected by mutation is:

(23) $$\begin{array}{c} {\left(1-{\mathrm{Pr}}_{m}\right)}^{\delta \left(z\right)}\text{.} \end{array}$$

## 4. Results and Discussion

The proposed methodology was implemented in the simulation environment and various experimental settings were performed.  Table 3 demonstrates the hardware and software needs for conducting the experiment.

### 4.1. Software and Hardware Description

The proposed fog-computing-based smart healthcare system is simulated with the help of iFogSim simulation tool. This tool is perfect for modelling the proposed work with the support of all possible topologies and scenarios.

Patient data is managed on the MySQL, and the same data is used to train the prescribed classifier. The data of 100 users was considered in the simulation where data is collected through various biomedical sensors.

### 4.2. Experimental Settings

**Figure 3:**
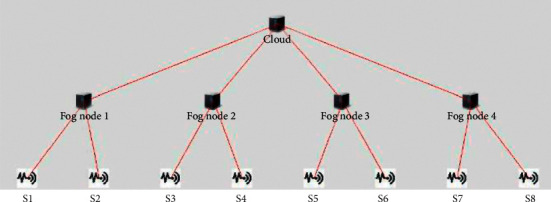
iFogSim simulation snapshot.

Table 4 shows the experimental settings for implementing the proposed smart healthcare system.  Figure 3 shows the simulation snapshot of 4 fog nodes. Simulation is extended up to 10 fog nodes.

### 4.3. Comparative Analysis on the Basis of Various Parameters

Various parameters were chosen for the comparative analysis such as latency, execution time, accuracy, and system stability. Various subsections following this section performed the required analysis on the acquired results. For comparative analysis, various existing systems are chosen from the literature survey. Fog-BBN [15], Fog-Smart Office [16], Fog-CPE [20], Tri-Fog Health [21].

#### 4.3.1. Latency Analysis

Latency is defined as the time taken for responding to the submitted request on the fog node. It is denoted by the following equation which includes the propagation and execution time.

(24) $$\begin{array}{c} l=t_{p}+e\text{.} \end{array}$$

Here, *t*_p_ represents the propagation time, and *e* is the execution time.

**Figure 4:**
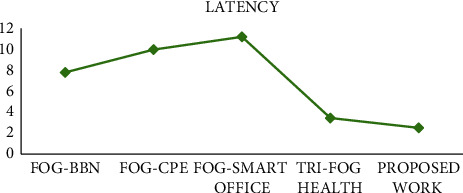
Latency comparison.

Figure 4 shows the latency comparison of various existing fog-based systems. Due to the preliminary steps performed before going into the fog layer, lesser latency could be observed in the proposed work. Workload optimization has significantly worked in the favour of reducing latency, consequently improving the overall system performance. State of the art methods are lacking load optimization, hence more delay can be observed.

#### 4.3.2. Analysis of Execution Time

**Figure 5:**
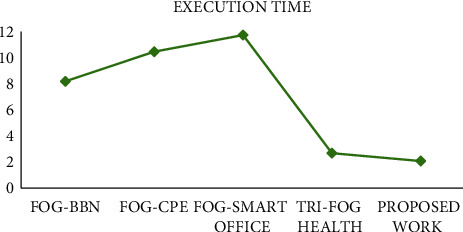
Comparison of execution time.

Execution time (in milliseconds) with respect to the healthcare system is the total time taken to decide whether the submitted request is critical or non-critical. Based on the criticality of the submitted request, it will be forwarded for next action.  Figure 5 shows the comparison of execution time for various existing fog-based systems. Optimization techniques, classification of task criticality, and noise removal process implemented in the fog layer have contributed to speed up the execution process. IoT applications require faster task execution, so that speedy results could be extracted for further processing. So, the proposed work has catered this requirement.

#### 4.3.3. Overall System Accuracy

**Figure 6:**
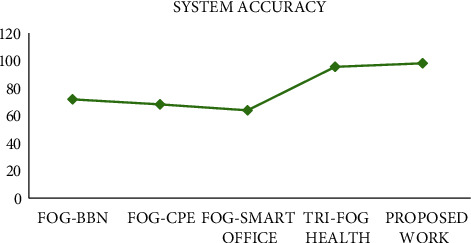
Overall system accuracy.

Comparison on the basis of overall system accuracy (in terms of %) was also performed. Accuracy is measured in terms of valid requests submitted to the fog nodes, true alerts for the critical tasks, and cleaned data for training the system. Keeping all these factors in view, Figure 6 shows the accuracy comparison for various existing fog-based systems, and the proposed work is overpowering all of them. State of the art methods are not performing sensor data cleaning through some preprocessing technique, hence suffering a lower system accuracy. Data analysis is another important factor here which can be managed properly. The proposed methodology is taking care of these aspects efficiently.

#### 4.3.4. System Stability

**Figure 7:**
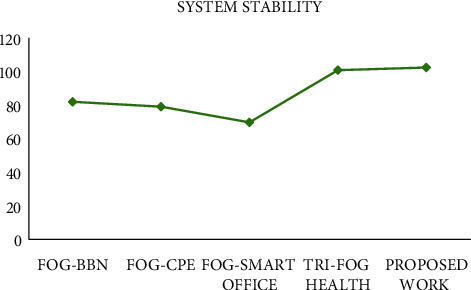
Comparison of system stability.

This parameter determines the scalability of the system and its ability to handle increasing number of users. Simulations were conducted on a large number of users (i.e., 100), and stability factor was determined for the same.  Figure 7 highlights the capability of the proposed work in terms of handling large number of users. Improvement in the other three performance metrics has certainly made sure that the system can work appropriately with the increase in the number of users.

### 4.4. Summary of Results

Comparative analysis mentioned in section 4.3 is clearly depicting the efficiency of proposed work in the field of healthcare systems.  Table 5 depicts the summary of the results gathered after performing the simulations.

Proposed work has shown considerable amount of improvement as compared to the recent and optimized literature work, i.e., Tri-Fog Health [21]. There is a percentage decrease of 27% in latency metric. Execution time drops by 23%, which shows the utility of proposed work. System accuracy increases by a factor of 2.57% and system stability is improved with a measure of 1.54%

## 5. Conclusions and Future Scope

In the proposed work, a novel framework for smart healthcare system is presented. Fog computing paradigm works closer to the IoT layer, so that processing could be done at the edges only and speedy response could be given. The presented methodology is implemented with the same concept. Fog layer could experience some delays and sometimes lack in the performance, as resource constraints are there. Presented framework tries to work on the same aspects and noticeable results were obtained. Variety of activities were also performed to achieve refinements in the results. This work could be extended to work in various other domains such as: Smart cities, Smart Homes, Smart Grides. These domains involve such activities which are time critical, and significant delays are not tolerable. Along with different domains, work could be done to introduce deep learning concepts for imparting better learning to the system. Scalability is another factor which is required to be improved, as in real-time scenarios, we may get more number of users, and a large amount of data is required to be processed.

## Figures and Tables

**Algorithm 1:**
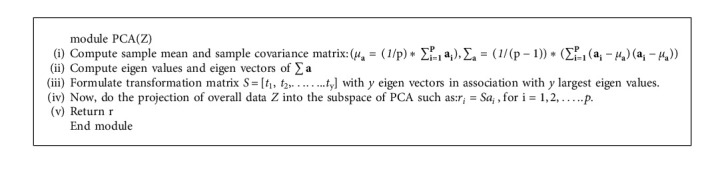
Principal component analysis for dimensionality reduction [23].

**Algorithm 2:**
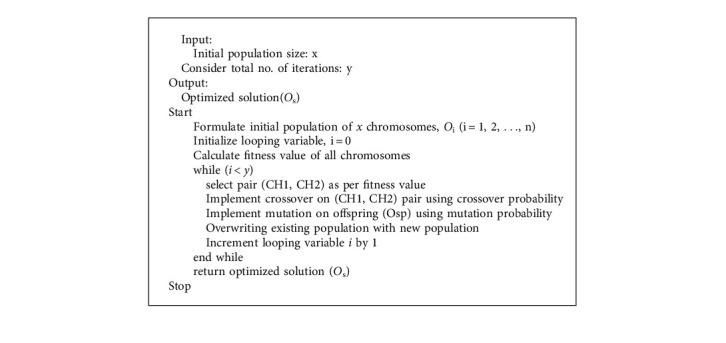
Genetic algorithm for workload optimization

**Table 1:** Strengths and limitations of related work.

Presented work	Strengths	Limitations
HealthFog [5]	Enhancement in Quality of service and prediction accuracy	(i) It could only work on single domain of diseases.
		(ii) Proposed architecture is designed for one application area only
STPH [6]	Efficient medical services	(i) Higher computational complexity
		(ii) Lack of medical offering system
HAAL-NBFA [7]	(i) Implementation of safe-fail module	Proposed framework can monitor limited set of diseases
	(ii) Minimum feature selection	
Fog-BBN [15]	Provides assistance for remote patient health monitoring	(i) Event severity level checking is missing
		(ii) Requirement of real time alert generation
Fog-Smart Office [16]	Severity Index calculation which reflects the impact of various activities in smart office environment	(i) Transmission of varied types of events in a common and adaptable format
		(ii) Lesser network load efficiency
Fog-CPE [20]	Minimization of time delay	Bi-directional coordination is missing
Tri-Fog [21] Health	(i) Elimination of faulty data	(i) Proposed system does not work on specific set of diseases (ii) Security aspects are not covered
	(ii) Removal of redundant data	
	(iii) Data processing using various attributes	

**Table 2:** Layer-wise functionalities and techniques.

Layer	Functionalities	Techniques/Resources
IoT layer	(i) Data collection	(i)Biomedical sensors
	(ii) Data Preprocessing	(ii) Noise removal and data cleaning
	(iii) Dimensionality reduction	(iii) Principal component analysis
Fog layer	(i) Task classification	(i) Support vector machines
	(ii) Workload optimization	(ii) Genetic algorithm
Cloud layer	(i) Analysis of critical tasks	(i) VMs, PMs

**Table 3:** Software and hardware description.

Software/Hardware	Description
Simulation tool	iFogSim
Simulator Version	3.0.3
Operating system	Windows 10
Programming Language	Java
JDK version	Java SE 12
IDE	Eclipse IDE 2021-03
Database	MySQL 8.0.24

**Table 4:** Experimental settings.

Parameter	Sub-parameter	Value
Number of users		100
Count of biomedical sensors		10
Count of fog nodes		10
Fog node configuration	Storage	2 GB
	Bandwidth	1500 KBs
	Resource cost	3.0-2.5
	Memory cost	0.5-0.4

**Table 5:** Results summary.

Parameter	Fog-BBN	Fog-CPE	Fog-Smart Office	Tri-Fog Health	Proposed Work
Latency (ms)	7.8	9.98	11.2	3.44	2.5
Execution Time (ms)	7.8	9.98	11.2	2.52	1.94
System Accuracy (%)	72.4	68.8	64.6	95.44	97.9
System Stability (%)	78.8	76	67	97	98.5

## Data Availability

The data presented in this study are available on request from the corresponding author.
